# Special Issue “Synthesis, Properties and Applications of Polymers”

**DOI:** 10.3390/ijms262010147

**Published:** 2025-10-18

**Authors:** Dmitriy A. Sapozhnikov

**Affiliations:** A. N. Nesmeyanov Institute of Organoelement Compounds, Russian Academy of Sciences, Vavilov Str. 28, Moscow 119334, Russia; ssddaa@ineos.ac.ru

This century, similarly to the twentieth century, is rightfully called the “Plastic Age”. Due to the wide variety of properties, including their ability to be tuned for specific practical applications, polymers have been able to take rightful place in all application areas. Despite the obvious problems with pollution [1,2], the production of polymers and the range of their applications are steadily expanding. Today’s world is impossible to imagine without polymers. Among all the applications, it is worth noting the significant role of polymers in such high-tech fields as medicine [3,4,5,6,7,8,9], energy [10,11,12], and optics [13,14,15]. All these application areas are considered in this Special Issue, entitled “Synthesis, Properties and Application of Polymers”, housing one review article and four original research articles.

Biopolymers have proved their worth in medical applications, including in tissue engineering, drug delivery, and regenerative medicine [16,17,18,19,20,21]. Chinese scientists have presented a comprehensive review of the latest developments in the use of chitosan-based polymers for peripheral nerve regeneration [22]. Chitosan-based polymers are notable for their biocompatibility, biodegradability, and ability to stimulate cell proliferation, making them highly suitable for eliminating nerve defects. They can form three-dimensional scaffolds that provide support and direction for nerve cells, ensuring an antibacterial effect. The porous structure of chitosan facilitates the transport of nutrients and oxygen, which leads to accelerated cell growth. Moreover, chitosan interacts with growth factors and cytokines, stimulating proliferation and regulating the process of nerve regeneration (Figure 1). This review article examines various techniques used to repair peripheral nerve injuries. In particular, attention is paid to neural epithelial suture, nerve grafting, nerve transfers, tubular conduits, nerve allograft, and tissue engineering conduits. The methods of tissue engineering, which include the use of hydrogels, 3D printing, and electric spinning, are discussed in detail. A comparative analysis of the prevalence; applicability; and limitations, advantages, and disadvantages of each method is performed.

A separate chapter is devoted to the biocompatibility, antimicrobial properties, porous structures, and loading factors of chitosan-based polymers. The key role of all these features for nerve injury repair is noted. A detailed analysis of literature sources dealing with the doping of various cell types with chitosan-based biopolymers (for example, adult neural stem cells, human embryonic stem cells, induced pluripotent stem cells, peripheral neural stem cells, etc.) for different applications is presented. Moreover, conduits made of chitosan-based polymers can be doped with biologically active molecules. These molecules include nerve growth factors, cell adhesion molecules, and drugs that play an important regulatory role in the regeneration of the nervous system. All these issues are analyzed in detail in this review as well.

Thus, chitosan-based polymers are multifunctional objects that can be used for nerve regeneration using various technologies. Their unique properties and versatility make them promising for regenerating peripheral nerve functions.

Polish scientists conducted a study on the comparative transplantation of autogenous and allogenic chondrocytes immobilized on polyethersulfone (PES) scaffolds for cartilage regeneration [23]. Three-dimensional, porous PES membranes made by the wet phase inversion method were used as a scaffold. The authors performed in vivo and in vitro studies. The main objective of this investigation was to evaluate the chondrogenic potential of chondrocyte transplants cultured in vitro on PES membranes (Figure 2).

To obtain more reliable results, 96 knee joints from forty-eight rabbits were used in the project, with the samples being divided into four groups: group I, wherein autogenic chondrocytes on PES membranes were transplanted into the defect area; group II, wherein allogenic chondrocytes on PES membranes were transplanted into the defect area; group III, wherein pure PES membranes were transplanted into the defect area; and group IV, wherein lesions were left untreated. The conducted full-scale studies led to the finding that the best cartilage regeneration was observed in rabbits from groups I and II.

In group I, where restoration was carried out using autogenic chondrocytes, such results were achieved after two operations, while for group II, in which allogenic chondrocytes were applied, only one operation was required. This indicates that cartilage repair using allogeneic chondrocytes has significant advantages and highlights the importance of further research to better understand the effects observed. The authors attribute the success and effectiveness of the PES scaffolds to the presence of sulfone groups in the polymer structure, similar to chondroitin sulfate, likely providing a mutual affinity between the scaffold and cells, thus facilitating the settlement of cells on the scaffold.

Dan Lu et al. present results on the conformational behavior of a ring-shaped polyelectrolyte in solutions of tetravalent salts, calculated using molecular dynamics simulations [24]. The effect of salt concentration and internal chain rigidity on the conformation of a ring-shaped polyelectrolyte in solution was studied in detail. By varying these parameters, it was established that the morphology of the ring-shaped polyelectrolyte and the self-assembly process can be controlled. When a flexible-ring polyelectrolyte is replaced by a semi-flexible one, the number of phase transitions changes from three to four. It has been established that the structural changes are caused by the strong interaction of the cations with the semi-rigid-ring polyelectrolyte. To simplify the calculations, the authors neglected the effect of twisting interactions, although it is well known that both bending and twisting interactions play a crucial role in the steric conformation of a semi-flexible-ring polymer. Additionally, to emphasize the rigidity of the ring polyelectrolyte, other properties were ignored. Nevertheless, the results obtained are useful for improving our understanding of the conformational behavior of charged biopolymers.

Lithium-ion batteries are well known and widely used, and their developers—J.B. Goodenough, M. S. Whittingham, and A. Yoshino—were rightfully awarded the Nobel Prize in Chemistry in 2019. However, most lithium-ion batteries pose a serious risk due to the reactivity of lithium and the flammability of liquid electrolytes. The development of safe, solid-state, mechanically durable polyelectrolytes with high conductivity and stability, as well as commercial appeal, is an urgent task [25,26,27,28,29,30]. In [31], the above-mentioned problems are solved by mixing imidazolium-containing acrylate-based polyionic liquids with different alkyl-spacers between the imidazoline fragment and the main chain, lithium bis (trifluoromethanesulfonyl)imide, and poly(propylene carbonate). A dual effect of using poly(propylene carbonate) (PPC) was discovered. PPC inclusion leads to an increase in both the mechanical strength of the composite and its ionic conductivity. Composite membranes have a conductivity of 10^−6^–10^−5^ S·cm^−1^ at room temperature, which can be increased to 10^−4^–10^−3^ S·cm^−1^ by adding 8–30 wt. % acetonitrile. The results of cyclic voltammetric measurements prove the good electrochemical stability of the membranes, including those saturated with acetonitrile. However, in real conditions, the decomposition of acetonitrile is expected. In future studies, the authors plan to use more stable plasticizers to address this issue.

Photopolymerization is a powerful tool widely used in dentistry [32,33], regenerative medicine [34,35], drug delivery [36], coating manufacturing [37], 3D/4D materials [38,39,40], etc. [41]. The excellent solubility of highly fluorinated cardo copolyimide (FCPI) based on 2,2-bis-(3,4-dicarboxydiphenyl)hexafluoropropane dianhydride; 9,9-bis-(4-aminophenyl)fluorene; and 2,2-bis-(4-aminophenyl)hexafluoropropane (1.00:0.75:0.25 mol) in various vinyl monomers and subsequent in situ polymerization made it possible to develop new materials with a range of valuable properties [42,43,44,45]. The authors of [46] present results obtained from studying the effect of FCPI on the photopolymerization kinetics of 1,4-butanediol diacrylate (BDDA); 1,4-butanediol dimethacrylate; ethylene glycol dimethacrylate; tetraethylene glycol diacrylate; triethylene glycol dimethacrylate; and tetraethylene glycol dimethacrylate. They examined the viscosity of photopolymerizable compositions and the properties of the resulting molecular composites as well. It was found that the viscosity of the solution and the kinetics of photopolymerization are affected by the nature of the monomer (diacrylate or dimethacrylate), the length of the spacer, and the concentration of FCPI. A study of the mechanical properties of composite films revealed a significant increase in their strength compared to unmodified carbon-chain polymers. Thus, the introduction of 23 wt.% FCPI into BDDA allowed for an enhancement of the tensile strength of the corresponding polymer from 20.2 to 44.0 MPa (2.2 times), an increase in the elongation at break percentage from 1.6 to 3.6% (2.2 times), and an increase in the modulus of elasticity from 1.5 to 1.9 GPa (1.3 times). The significant change in mechanical strength is due to the formation of both non-covalent and covalent bonds between FCPI and the three-dimensional network [42,47,48,49,50]. The decomposition temperatures of composites increase by 10–30 °C compared to unmodified carbon-chain homopolymers. Two of the most promising photopolymerizable compositions were tested in the formation of primary protective coatings of silica optical fibers [51], which are widely used in medicine, energy, etc. [14,52,53,54,55,56]. It was found that the new coating based on poly(BDDA–FCPI_23%_) could withstand prolonged annealing at 200 °C (72 h), which is comparable or superior to the known most thermally stable photo-curable coatings. This approach is promising not only for creating highly thermostable coatings but also for other functional products, such as 4D materials with shape memory effects [45].

This Special Issue features a review article and four original research articles on the applications of polymers in medicine, electrochemical devices, and optics. Despite the limited number of papers included, they demonstrate the diverse possibilities of using polymers in the most advanced fields.

## Figures and Tables

**Figure 1:**
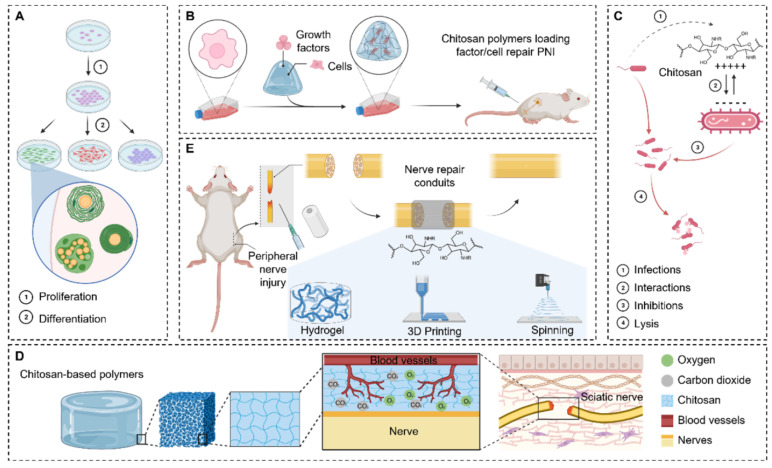
Chitosan-based biopolymer repair of a peripheral nerve injury. (**A**) Chitosan-based polymers have excellent biocompatibility. (**B**) Chitosan-based polymer materials can act as carriers of loaded cells and factors to promote peripheral nerve regeneration. (**C**) Chitosan has excellent antibacterial properties. (**D**) The porous structure of chitosan-based polymers facilitates vascular regeneration and the exchange of oxygen and nutrients. (**E**) Patterns of peripheral nerve injury treated with chitosan-based biopolymers (reproduced with permission [22]).

**Figure 2:**
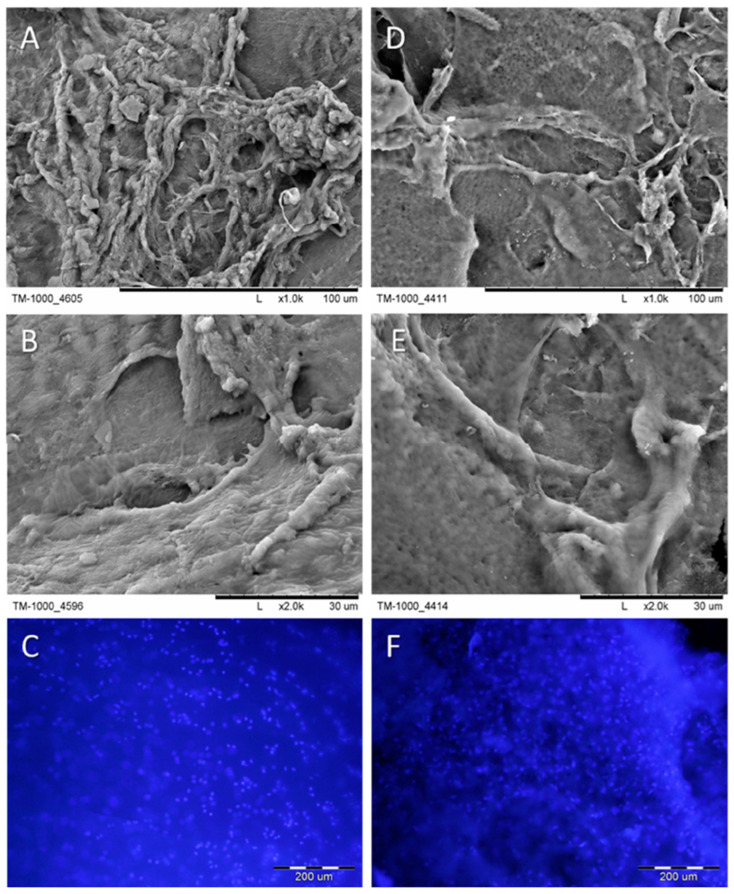
SEM micrographs of native cartilage and recoveries (cells with their products) were obtained from scaffolds after 6 weeks of culture. Samples were stained with Hoechst dye to visualize cell nuclei. (**A**–**C**)—native cartilage; (**D**–**F**)—recoveries from PES scaffold. Scale bars: (**A**,**D**)—100 μm; (**B**,**E**)—30 μm; (**C**,**F**)—200 μm (reproduced with permission [23]).
